# Design and Fabrication of the Split Ring Resonator Shaped Two-Element MIMO Antenna with Multiple-Band Operation for WiMAX/5G/Zigbee/Wi-Fi Applications

**DOI:** 10.3390/mi13122161

**Published:** 2022-12-07

**Authors:** Ammar Armghan, Khaled Aliqab, Vishal Sorathiya, Fayadh Alenezi, Meshari Alsharari, Farman Ali

**Affiliations:** 1 Department of Electrical Engineering, College of Engineering, Jouf University, Sakaka 72388, Saudi Arabia; 2 Faculty of Engineering and Technology, Parul Institute of Engineering and Technology, Parul University, Waghodia Road, Vadodara 391760, India; 3 Department of Electrical Engineering, Qurtuba University of Science and IT, Dera Ismail Khan 29050, Pakistan

**Keywords:** MIMO, 5G communication, antenna, gigahertz, gain, bandwidth, WiMAX, Wi-Fi

## Abstract

In this manuscript, we proposed the split ring resonator loaded multiple-input multiple-output (MIMO) antenna design for the frequency range of 1 and 25 GHz. The proposed antenna is numerically investigated and fabricated to analyze the different antenna parameters. We provided statistics on a wide range of antenna parameters for five different designs, including a simple circular patch antenna, a single-split-ring antenna, and a double-split-ring antenna. Reflectance, gain, directivity, efficiency, peak gain, and electric field distribution are all analyzed for all proposed antennas. The maximum achievable bandwidth is 5.28 GHz, and the double-split-ring resonator structure achieves this with a return loss of −20.84 dB. The radiation patterns of all the antenna with different port excitation conditions are presented to identify the behavior of the antenna radiation. We found the effect of the split-ring resonators to form radiation beams in different directions. We found the maximum and minimum half-power beam widths of 75° and 2°, respectively, among the different antenna designs. It was found that the split-ring resonator geometries in patch antenna convert wide-beam antenna radiation patterns to several narrow-beam radiation patterns. We found that each antenna’s bandwidth, gain, and return loss performance significantly differs from the others. Overall, the proposed results of the antenna may apply to a wide range of communication applications, including those for Wi-Fi, WiMAX, and 5G.

## 1. Introduction

The current prospective technologies help to try high data rates and low-priced applications. In line with their wideband systems can rapidly transfer a great deal of data across a wide variety of frequency channels while using a relatively low power spectral level [1]. There has been a tremendous shift in wireless communication networks over the past decade, yet the demand for compact, portable devices has remained steady. New wireless networks aim to accommodate many users simultaneously, providing variable data speeds and a versatile set of services. However, spectral congestion limits the available bandwidth, a barrier to increased data rates. Therefore, antenna solutions that allow integration across several frequency bands are becoming increasingly crucial for wireless devices [2]. Already in many smartphones and other portable devices, they may soon find broader application in consumer electronics [3]. Multi-input, multioutput (MIMO) technology is also employed to reduce multipath fading and expand available channel capacity [4,5]. MIMO enters the scene to increase system capacity and dependability. Multi-input multioutput (MIMO) relies on a large number of independent antennas for both transmission and reception. Different metrics can assess antenna array performance in MIMO systems [6,7].Using multiple-input multiple-output (MIMO) systems with multiple antenna modules at both the transmitter and receiver may improve wireless systems’ multipath performance [8]. With MIMO technology, all antennas must be active at the same time.

The research shows that when the MIMO structure gets smaller, the isolation between the antenna components also decreases [9]. With the help of a radial stub-loaded resonator, MIMO antennas consisting of two antenna components have been constructed [10]. A flawed isolation wall is used to probe methods for decreasing mutual coupling, as presented in [11]. The results of the antenna presented in [12] demonstrate that isolation and bandwidth are sufficient, but they do not consider the antennas’ diversity characteristics. Slotted triangular parasitic patches and two PIN diode switches [13,14] are used in MIMO to increase the principal triangle radiator’s electrical length, enabling triple-band capabilities. When trying to achieve sub-6 GHz applications, the diagonally trunked patch with the partial diffracted ground concept is a useful tool, as depicted in the article [15]. Using an L-shaped slot in the radiating element and two identical stubs coupled to the partial ground improves the broadband radiation characteristics and impedance matching throughout the bands of interest [16]. In [17], a multi-input multioutput (MIMO) antenna based on four monopole antennas is proposed, enabling communication at mm-wave and sub-6 GHz frequencies while maintaining high isolation. An extensive and detailed pattern is conceivable because of the two layers of the mushroom structure. The inverted L-monopole construction [18] allows the four-element wideband MIMO antenna to cover an extensive frequency range. However, the achieved isolation is just −11 dB across the usable frequency range. There is much interference observed in the proposed antenna [19] because of the inadequate isolation between the antenna sections. When an antenna is constructed from a single element, the center frequency changes if a common ground is used and if the components are not well-isolated from one another [20]. In [21], the author proposes using a two-element MIMO antenna that has band-notch characteristics. The antenna is relatively large, but its strength is only 2 dBi. The two-element MIMO antenna presented in [22] has a wide operating frequency range of 2.1–11 GHz. On the other hand, we only managed to achieve a gain of 4 dBi and isolation of −22 dB. The machine learning capability of the MIMO antenna allows it to be used on a broader variety of settings. Federated learning (FL) is a machine learning form that considers privacy concerns by having users train models locally before sending their tweaks to a central server. Since data owners are not required to participate in FL, it is a privacy-invasive machine-learning approach [23,24]. In sum, these characteristics facilitate the realization of IoT-based applications. Interest in the next-generation 5G heterogeneous networks is widespread. There are huge benefits and many worries associated with using the bandwidth spectrum of HetNets to transfer massive amounts of data at rapid speeds [25]. Creating a reliable 5G antenna design is a key step toward maximizing the potential of the 5G network [26]. A transparent MIMO antenna made from a plexiglass base and transparent conductive oxide is described in the paper [27]. The proposed MIMO design for state-of-the-art 5G technology in [28] takes a novel method to increase isolation between several antenna components. Using two quasi-self-complimentary MIMO antennas to achieve broad bandwidths is proposed in [29]. This antenna is perfect because of its high gain and compact size. Due to its position, a lower ECC and lower isolation than other portions of the system are present in the quasi-structure. In the 2.3–2.4 GHz operating range, up to −40 dB isolation can be attained using the mushroom structure in a four-element MIMO antenna [30]. Massive MIMO is a frontrunner for the 5G network due to its great spectral efficiency and low power consumption. However, pilot contamination is a major barrier for huge MIMO systems (PC). Thus, a two-pilot scheduling approach is analogous to a synchronous fractional pilot scheduling system (AFPS) and a fractional pilot reuse (FPR) [31].

The proposed antennas in the previous literature survey provide a limited scope of the antennas’ performance with specific gain and isolation properties. The bandwidth properties for the wide range of GHz applications are also limited in previously designed antennas. Therefore, it is essential to cover a wide range of GHz bands through the single-antenna design, which offers better isolation, wideband, and improvised gain. It is also required to design an antenna which can be worked on multiband applications for a wide range of device adaptability. Inspired by the previous antenna design and its application in wireless communication, we designed the MIMO antenna, which offers wideband operation with minimum return loss values and high radiation gain. We also designed an antenna that offers multiband operations and can work on narrow-band operation for specific antenna radiation applications. Overall, the antenna design proposed in this research can be applied in wideband, short-band, and multiband operations.

We simulated and fabricated a dual-element MIMO antenna structure for the 1 to 25 GHz operating frequency band. The proposed antenna is analyzed with different antenna patch structures, which include the circular patch, single split-ring-engraved and double split-ring resonator based on radiating elements. We investigated various antenna parameters such as return loss, gain, bandwidth, directivity, radiation pattern, efficiency, and normalized electric field to identify the overall behavior of the antenna. The proposed antenna can offer multiband resonating behavior for the 1 to 25 GHz of the frequency band.

## 2. Split Ring Resonator Loaded MIMO Antenna Structure

**Figure 1:**
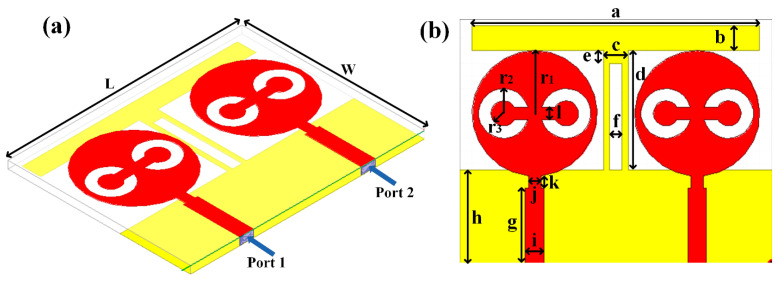
(**a**) Schematic of the split ring resonator loaded MIMO antenna. (**b**) The top (red) and bottom (yellow) layer structure is considered conductive copper material on FR4 substrate.

A split ring resonator based two-element MIMO antenna structure is numerical and experimentally analyzed over the 1 to 25 GHz frequency range frequency spectrum. The schematic of the proposed antenna is shown in Figure 1. Figure 1a shows the perspective view of the antenna. Figure 1b shows the top and bottom view of the antenna with the notation of the dimensions. The values of each dimension are shown in Table 1. The overall size of the antenna is set as 39 × 50 mm^2^.

We fabricated the five antenna structures to identify other design parameters and shapes. These antenna and its description are shown in Table 2. These antennas have two circular patch-shaped designs where single and multiple split rings are engraved to identify the effect in return loss and radiation effect. These antennas are excited with the lumped plot element method with a rectangular port, as shown in Figure 1a. The FR4 sheet used to create the antenna is double-sided. The top and bottom layers are copper materials with perfect electric conductor properties. HFSS antenna modeling software was used to build the proposed structure. A squared patch is created by deducting the split ring from the required area. The top and bottom layers are ideal electric conductors with boundary conditions. Air is considered material when positioning the radiation box with the antenna. For the outer radiation box, we specify the radiation border condition. When the ground is used as a reference plane, the port depicted in Figure 1 is excited as a lumped port. The frequency sweep on the exciting port is what runs the simulation. Post-processing techniques are applied to the HFSS simulation of the antenna to extract the reflectance loss, gain, and directivity values.

## 3. Results and Discussion

**Figure 2:**
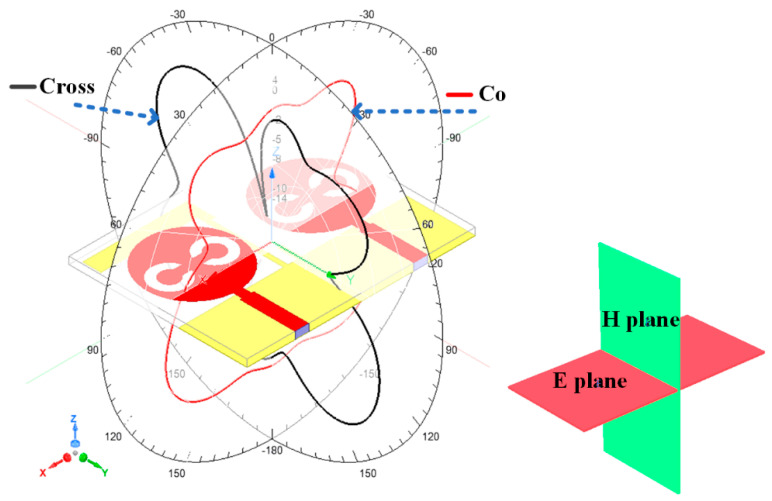
Representation of co−and cross−polarization plane to its axis system for design 4 antenna system with both port excitation conditions with E and H plane notation.

**Figure 3:**
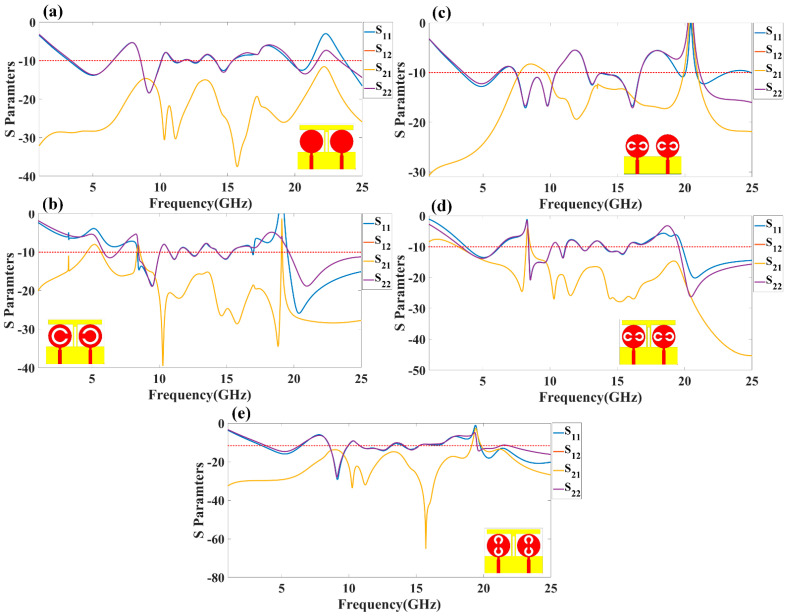
Calculated reflectance loss factors (S_11_, S_12_, S_21_, and S_22_) (in dB) for (**a**) design 1, (**b**) design 2, (**c**) design 3, (**d**) design 4, and (**e**) design 5 of the proposed structure of the antenna.

The proposed antenna structure is numerically investigated and measured over the 1 to 25 GHz frequency range. We analyzed the different terms of the antenna, such as return loss parameters, gain, directivity, radiation pattern, efficiency, and electric field distribution. Figure 2 shows the representation of the co- and cross-polarization plot to the axis notation for the design 4 antenna with both port excitation conditions. Figure 3 shows the return loss values of the different S parameters (S_11_, S_12_, S_21_, and S_22_) (in dB) for design 1 and design 2. The S parameters are referred to as the power transfer ratio from one port to another. For example, the S parameters’ term S_ij_ (i, j = 1, 2) refers to the ratio of the power transferred from jth port to ith port. Figure 3a shows the return loss values for design 1. The seven operating bands of the operation are observed in a simulation study. The top band of the operation is 3.9 GHz for the frequency range of 3.38 to 6.47 GHz. The return loss observed in this band is −13.81 dB. The minimum return loss of −18.41 dB in this antenna was observed between the 8.55 to 10.07 GHz frequency range. Figure 3b shows the return loss values for design 2. The five operating bands of 1.47 GHz, 0.66 GHz, 0.7 GHz, 1.06 GHz, and 5.38 GHz are observed in a simulation study. The maximum band of the operation is 5.38 GHz for a frequency range of 19.62 to 25 GHz. The return loss observed in this band is −25.91 dB.

Figure 3c–e shows the return loss variation observed in design 3, design 4, and design 5. Figure 3a shows the return loss values for design 3. The six operating bands of 2.54 GHz, 0.29 GHz, 0.73 GHz, 3.94 GHz, 2.18 GHz, and 0.4 GHz are observed in a simulation study. The maximum band of the operation is 3.94 GHz for a frequency range of 12.75 to 16.69 GHz. The return loss observed in this band is −17.13 dB. Figure 3d shows the return loss values for design 4. The simulation study observed six operating bands of 2.46 GHz, 1.57 GHz, 0.51 GHz, 0.68 GHz, 1.73 GHz, and 5.18 GHz. The maximum band of the operation is 5.18 GHz for a frequency range of 19.82 to 25 GHz. The return loss observed in this band is −26.29 dB. Figure 3e shows the return loss values for design 5. The four operating bands of 3.42 GHz, 1.66 GHz, 6.55 GHz, and 5.28 GHz from the simulation study are observed. The maximum band of the operation is 5.28 GHz for a frequency range of 19.22 to 25 GHz. The return loss observed in this band is −20.84 dB. The overall minimum return loss of −29.33 dB is observed in the second band of 8.45 to 10.11 GHz frequency band. In Figure 2 of the S parameters’ values of the antenna, the response of the S_12_ is overlapped with the parameter S_21_. The reason for overlapping both responses is the ideally mirrored image on both sides of the antenna over the center axis.

The detailed comparative values in the operating frequency, a band of operation return loss, and the number of operating bands in each antenna are shown in Table 3. All the values for calculating the band and operating frequency are considered, where all the S parameters are <−10 dB. It is observed from all the designs that the engraved shape of the split-ring resonator and stub generate different operating bands with different return loss values. This result allows choosing the MIMO shape variant for the specific antenna operating band. The operating band is changing because the electric field concentrates on the different shapes and edges.

**Figure 4:**
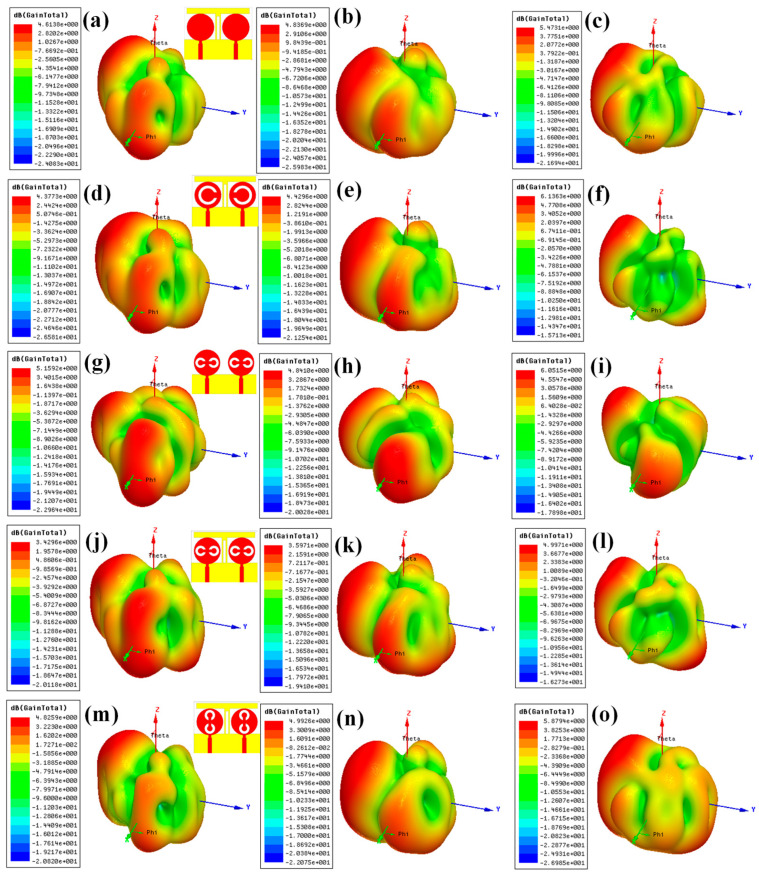
Variation in the radiation in 3D polar plots for the different port excitation conditions of (**a**–**c**) design 1, (**d**–**f**) design 2, (**g**–**i**) design 3, (**j**–**l**) design 4, and (**m**–**o**) design 5 antenna. The different port excitation conditions are (**a**,**d**,**g**,**j**,**m**) P_1_ = 0 and P_2_ = 1, (**b**,**e**,**h**,**k**,**n**) P_1_ = 1 and P_2_ = 0, and (**c**,**f**,**i**,**l**,**o**) P_1_ = 1 and P_2_ = 1.

**Figure 5:**
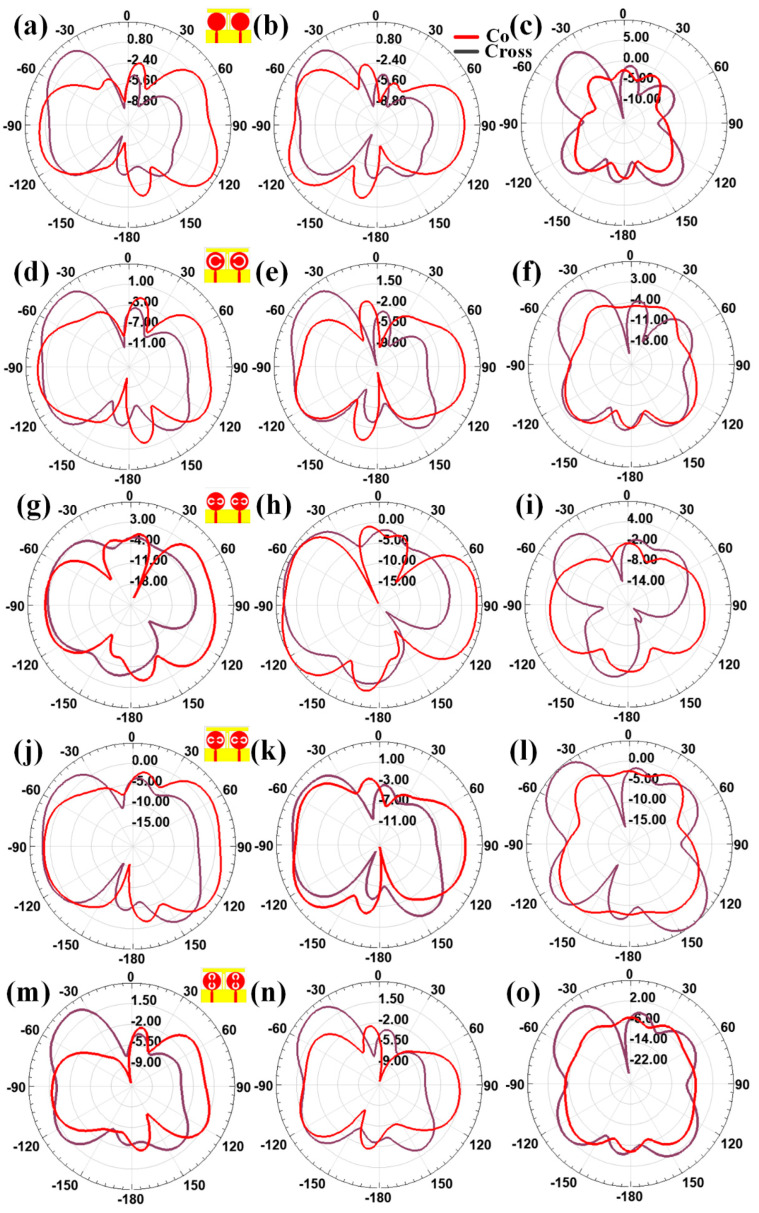
Variation in the radiation in 2D polar plots for the different port excitation conditions of (**a**–**c**) design 1, (**d**–**f**) design 2, (**g**–**i**) design 3, (**j**–**l**) design 4, and (**m**–**o**) design 5 antenna. The different port excitation conditions are (**a**,**d**,**g**,**j**,**m**) P_1_ = 0 and P_2_ = 1, (**b**,**e**,**h**,**k**,**n**) P_1_ = 1 and P_2_ = 0, and (**c**,**f**,**i**,**l**,**o**) P_1_ = 1 and P_2_ = 1.

Figure 4 shows the two-dimensional and three-dimensional radiation patterns for the design 1 antenna. Figure 4a,c,e shows the variation in three-dimensional radiation pattern for the different port excitation conditions of (P_1_ = 0 and P_1_ = 1), (P_1_ = 1 and P_1_ = 0), and (P_1_ = 1 and P_1_ = 1), respectively. Similarly, Figure 5a–c shows the variation in two-dimensional radiation pattern for the different port excitation conditions of (P_1_ = 0 and P_1_ = 1), (P_1_ = 1 and P_1_ = 0), and (P_1_ = 1 and P_1_ = 1), respectively. Similarly, Figure 4d–o shows the three-dimensional and two-dimensional radiation patterns for design 2, design 3, design 4, and design 5 of antennas. Notably, variation in the antenna radiation pattern is observed for the different port conditions along with the peak gain achieved. The >4 dBi gain is observed in all the antenna designs with different port conditions.

We calculated the half-power beamwidth of the different antenna for both port excitation conditions, and its comparative analysis is shown in Table 4. The beam width variation is observed from a narrow beam to a wide beam (HPBW), ranging from 2° to 75°. The comparative analysis is presented in Table 4 in terms of design, polarization conditions, and HPBM range. HPBM is calculated from the normalized bandwidth of the overall pattern to >−3 dB values, which are referred to as half-power range. It is observed from Figure 5a,b that the left-hand plane offers a wide beam width compared with the right-hand plane. As shown in Figure 5c and Table 4 (Design 1), the maximum and minimum values of HPBW are 44° and 7° in co-polarization conditions. In cross-polarization conditions, the maximum 36° of the HPBW is also observed in −63° to −27° of the radiation plane. In the radiation pattern of design 2 shown in Figure 5f, we observed a maximum HPBW of 42°. The addition of the split ring in the top MIMO patch increases the beam width of the directivity. Similarly, the wide beam width of 75° in the double split-ring resonator structure, as shown in Figure 5i for design 3 of the antenna structure, is observed. The effect of adding the stub in the bottom layer increases the radiation beam on the overall radiation patterns. The stub in the middle converts the majority of single-beam radiation to a multibeam radiation pattern. The effect of this multibeam radiation is observed in Figure 5l for design 4 of the antenna structure. The maximum and minimum beamwidths in the radiation pattern are observed as 62° and 5° in the co-polarization pattern. Similarly, the maximum and minimum beamwidths in the radiation pattern are observed as 37° and 2° in the co-polarization pattern. The double split-ring orientation change generates the maximum and minimum HPBWs of 46° and 3° for the co-polarization structure for design 5, as shown in Figure 5o. In the cross-polarization radiation pattern, the maximum and minimum HPBWs are 38° and 13°. The effect of the split ring is mainly observed for shaping the multibeam antenna radiation structure by applying specific ring orientation. The phase difference between co- and cross-polarization antennas is 0° when peak gain values are high. These conditions are observed for all port excitation conditions. It will make the antenna behavior be in linear polarization mode. This phase difference can be observed in Figure 4c,f,i,l,o.

**Figure 6:**
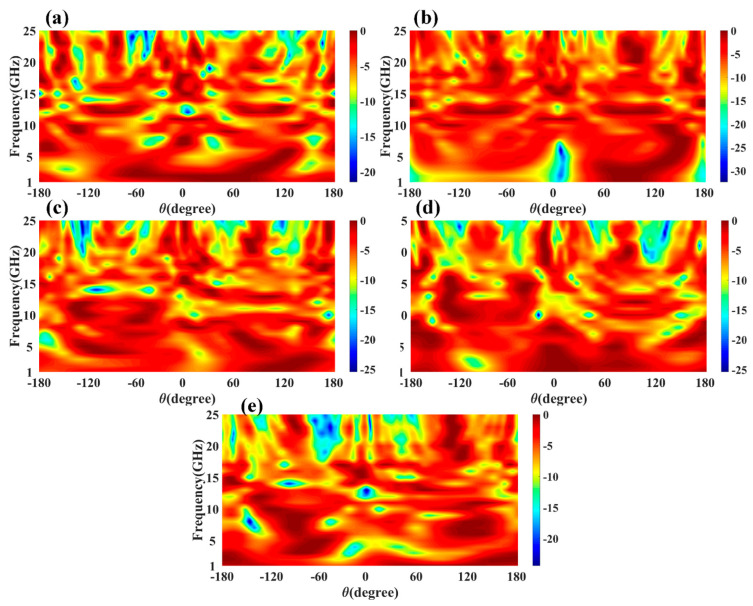
Calculated variation in normalized gain (dBi) for the different polar angles and frequency spectrums. Variation in gain for the different (**a**) design 1, (**b**) design 2, (**c**) design 3, (**d**) design 4, and (**e**) design 5 antennas.

**Figure 7:**
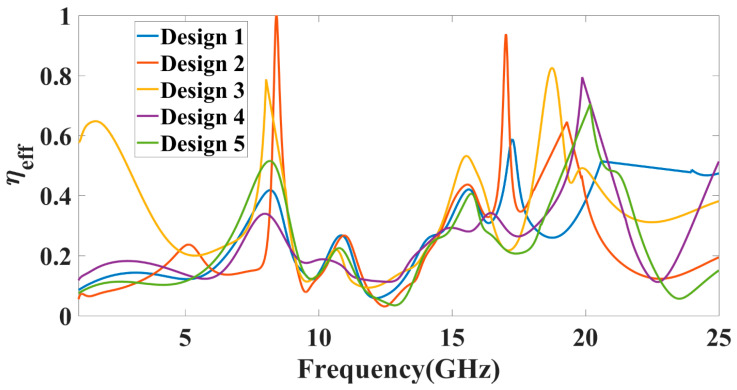
Variation in antenna radiation efficiency for different designs of antenna.

We also calculated the variation in directivity for the entire frequency spectrum band, and the results are shown in Figure 6. Figure 6 shows the change in normalized directivity (dB) for the angle values of −180° to 180° and phi of radiation pattern 0 degrees for the entire frequency spectrum of 1 to 25 GHz. We can also identify the values of the >−3 dB operating band for the specific radiation rather subgraph and its suitable frequency bands. Figure 7 shows the radiation efficiency of the different antenna designs over the entire frequency band of the operation. The variation in frequency excitation from 1 to 25 GHz values of this process gives us the efficiency values of the spectrum bands. The maximum radiation efficiency peak shows near the 8 GHz and 18 GHz frequency points. Comparing the return loss values and gain plot, these two points do not show the practical return loss values. In other bands of the operation, as shown in Figure 3, the average radiation efficiency is observed at > 40% for most bands. Therefore, we can choose the effective radiation efficiency, return loss values, gain, and directivity values to identify the effective operation of the MIMO antenna. The radiation efficiency allows for identifying the broad operating frequency band where the antenna radiated to its higher amplitude of electromagnetic radiation. The combined results of the return loss and efficiency allow for choosing the type of design, operating frequency spectrum, and radiation bandwidth.

**Figure 8:**
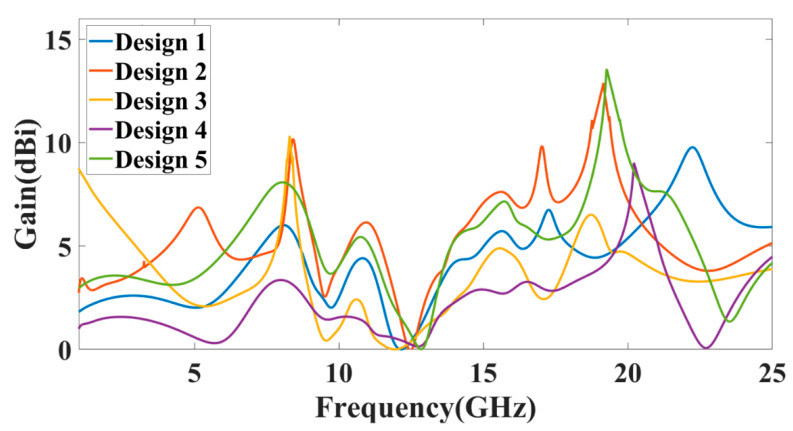
Variation in peak gain (dBi) for the different antenna designs.

**Figure 9:**
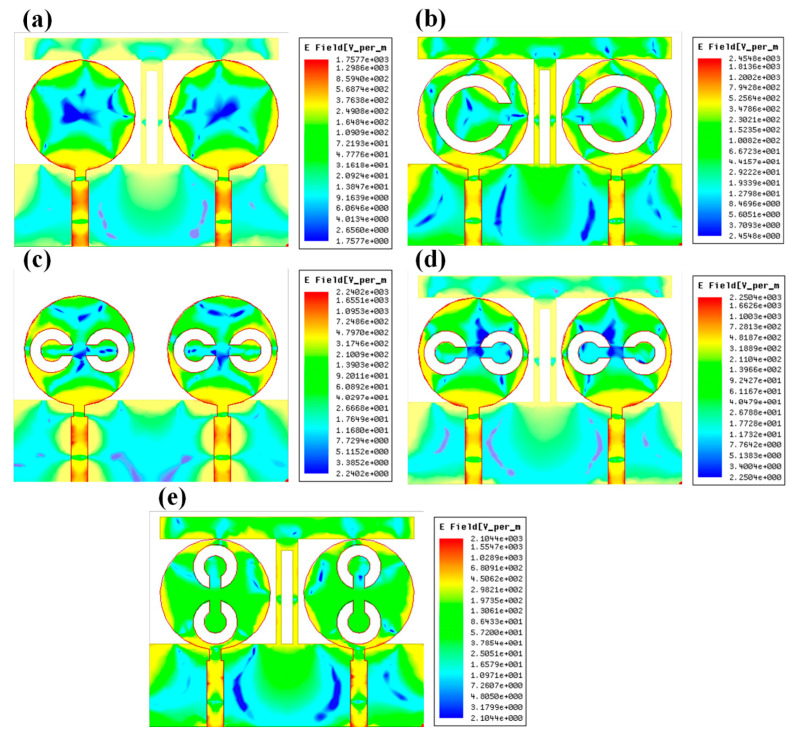
Normalized electric field distribution for the different shapes of the structures. The normalized electric field intensity for the different antenna designs (**a**) Design 1, (**b**) Design 2, (**c**) Design 3, (**d**) Design 4, and (**e**) Design 5 for the 10 GHz frequency value.

**Figure 10:**
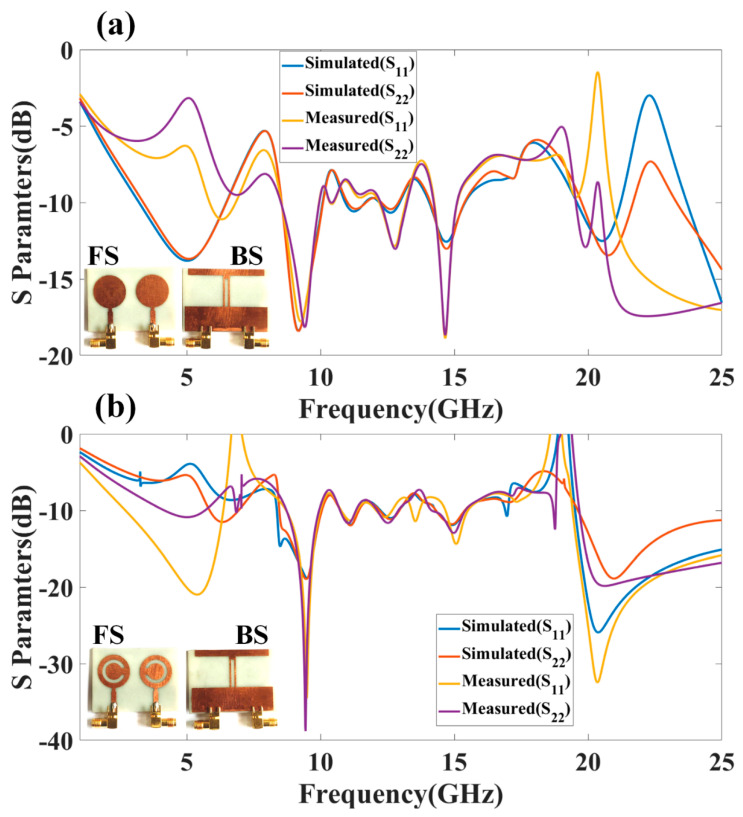
Comparative plot of simulated and measured reflectance loss factors (S_11_, S_22_) (in dB) for (**a**) design 1 and (**b**) design 2 of the proposed structure of the antenna.

**Figure 11:**
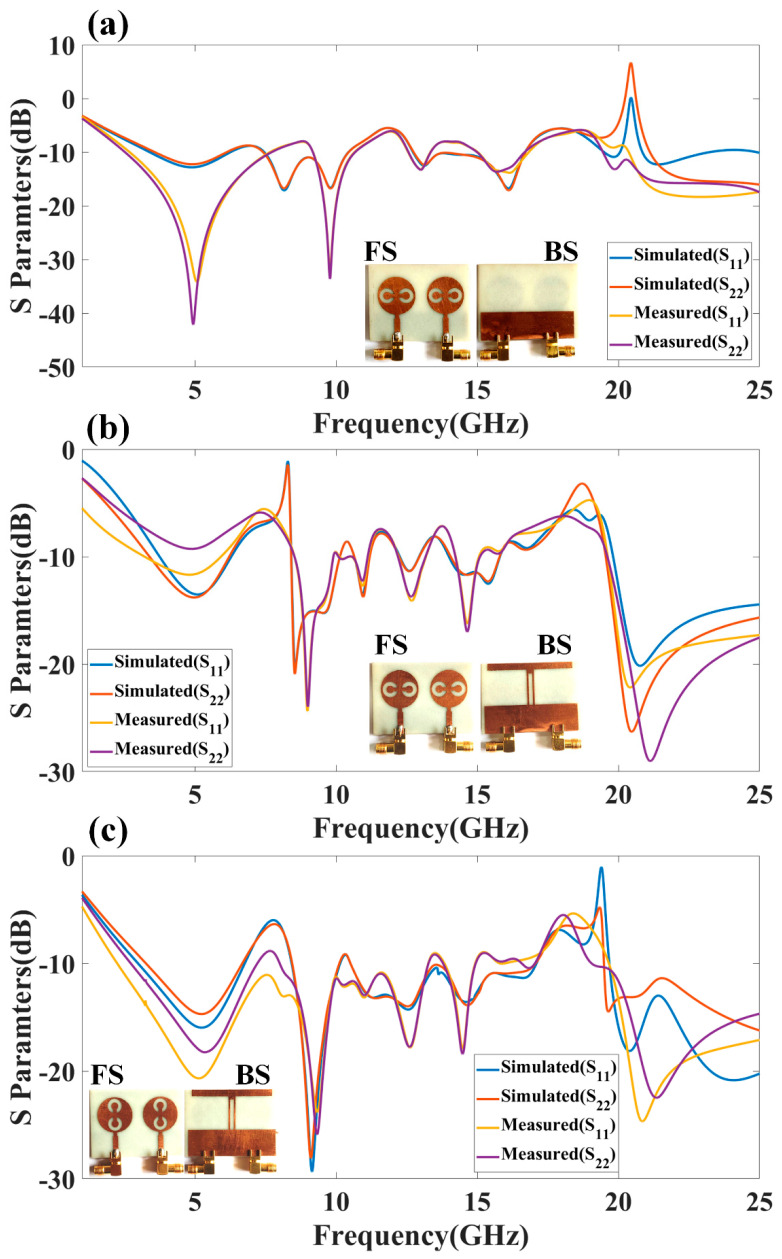
Comparative plot of simulation and measured reflectance loss factors (S_11_, S_22_) (in dB) for (**a**) design 3, (**b**) design 4, and (**c**) design 5 of the proposed structure of the antenna.

**Figure 12:**
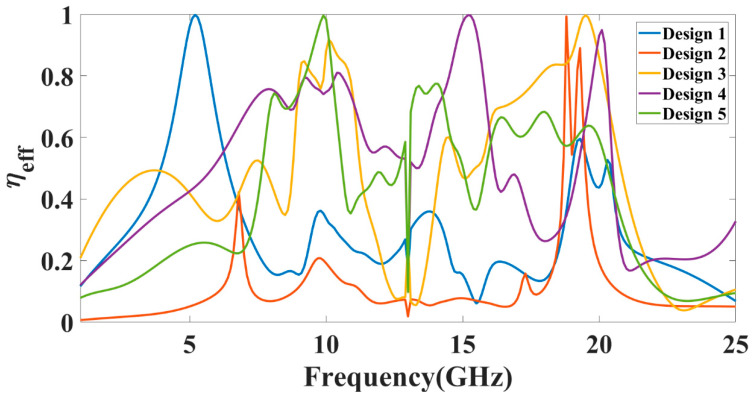
Variation in antenna radiation efficiency (measured) for different antenna designs.

**Figure 13:**
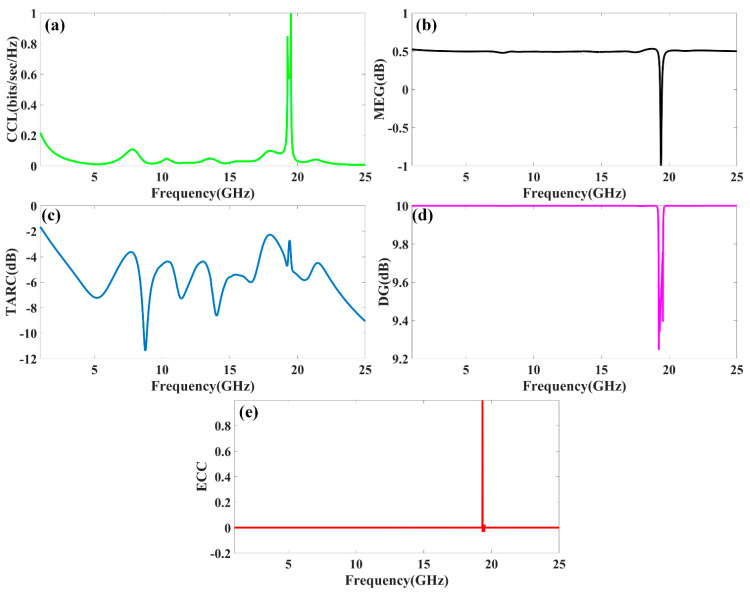
Variation in different MIMO antenna parameters (**a**) CCL, (**b**) MEG, (**c**) TARC, (**d**) DG, and (**e**) ECC for the design 4 antenna structure.

**Figure 14:**
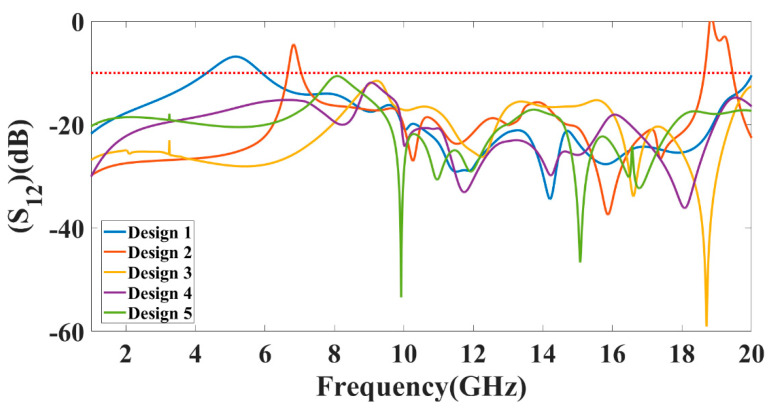
Comparative plot of measured cross-port reflection values (S_12_) for the different antenna designs.

The peak gain achieved in all the antenna ranges for the 1 to 25 GHz of the frequency spectrum is presented in Figure 8. It is observed from the combined return loss and gains spectrum that the peak gain is not the only factor in overall antenna operation that can justify. It is essential to look first for the fair return loss values, antenna efficiency, and gain value to judge the overall performance of the proposed antennas. In some bands, the return loss is >−10 dB, but gain values seem higher. The peak near to 20 GHz frequency values are >−10 dB. This combination is not helpful for effective antenna radiation. This type of condition leads toward the oscillating behavior of the antenna instead of radiating behavior. Figure 9 shows the different antenna designs’ electric field (V/m) distribution on radiation planes. It is observed that there are different field distributions over different shapes and engraved structures. Variation in the electric field distribution is changing per the split ring resonator based engraved design and substrate stub design. The electric field distribution over the different shape structures creates different resonator points of frequency. It will affect variation in reflectance parameters and gain values. Figure 10 and Figure 11 show the variation in the reflectance loss between simulated and measured results of the proposed antenna structures. This MIMO antenna design has a very small resonating point variation. The feed points are the primary ones responsible for the significant variation in the measured and simulated results for specific operating bands. The SMA connector adds a significant amount of loss to the measurements. The resonator’s bandwidth, however, is roughly the same in both cases. Measured and simulated results for these MIMO antennas show significant variation, which can be attributed in part to the effect of the soldering materials. Either result’s alteration may also be caused by the connector employed during the measurement set. The impedance of the connector or the antenna may cause this difference. The MIMO antenna parameters such as ECC (Envelope Correlation Coefficient), CCL (Channel Capacity Loss), MEG (Mean Effective Gain), DG (Directivity Gain), and TARC (Total Active Reflection Coefficient) [5,32,33,34] were investigated for the proposed structure 4 antenna. The achieved diversified MIMO parameter results of the proposed design 4 antenna show the values of ECC < 0.01, CCL < 0.5 bps/Hz/s, DG ≈ 10 dB, TARC< −10.0 dB, and MEG < 0.5 dB). It helps overcome the obstacles inherent in short-distance communication, such as fading signals, increased interference, and The measured antenna efficiency for the proposed antenna structure is shown in Figure 12. In the simulation, efficiency is measured by defaulted simulation setup and parameter extraction provided by the HFSS simulation package. The ratio of the received power and transmitted power extracts the efficiency of the measured antenna. multipath propagation. These MIMO antenna parameters for the design 4 antenna structure are shown in Figure 13. The comparative analysis of the proposed structure with the previously published results is shown in Table 5. The comparison of the proposed structure is derived in terms of the isolation, ECC, antenna structure area, antenna elements, and design complexity. Figure 14 shows the variation in cross-port reflectance loss values for the different antenna designs. The comparative analysis of the S12 values is majority <−10 dB in the whole spectrum for most antenna designs. This response suggests that all the antenna generates the minimum cross-port interaction as the values are <−10 dB.

## 4. Conclusions

We presented numerical and measured results of the split ring resonator loaded antenna for the 1 to 25 GHz frequency range. We showed the different antenna parameter results for the five antenna types, which contain the simple circular patch shape, single split-ring, and double split-ring resonator structures. The antenna is analyzed in terms of the different reflectance parameters, gain, directivity, efficiency, peak gain, and electric field distribution. The proposed antenna offers a maximum bandwidth of 3.09 GHz with −13.81 dB return loss in a normal circular patch antenna. In the case of the single split-ring resonator, we observed the 5.38 GHz bandwidth with −25.91 dB of return loss. The double split-ring resonator structure offers the maximum bandwidth of 5.28 GHz with −20.84 dB of the return loss. We observed the bandwidth, return loss, gain, and bandwidth variation for all antenna structures. The proposed antenna’s overall results can apply to various Wi-Fi, WiMAX, and 5G communication applications.

## Figures and Tables

**Table 1:** Antenna design parameters.

**Parameter**	a	b	c	d	e	f	g	h	i	j	k	l	W	L	r_1_	r_2_	r_3_
**Values (mm)**	46	4	4	19	2	2	12	15	3	2	2	3	39	50	10	4	2

**Table 2:** Different antenna designs and their descriptions.

Sr. No	Design	Description
1	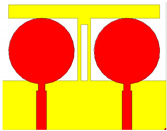	Circular patch with stub in the back layer
2	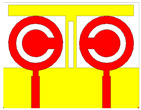	Circular patch with split-ring-engraved structure with stub in the back layer
3	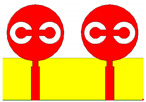	Circular patch with dual-split-ring resonator with a normal rectangular patch on the backside
4	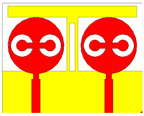	Circular patch with dual-split-ring resonator with stub structure patch on the back side
5	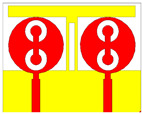	Circular patch with 90-degree-rotated dual-split-ring resonator with the normal rectangular patch on the backside

**Table 3:** The comparative study of achieved simulation results of frequency band and minimum return loss for the proposed antennas.

Design	$f_{min}\;(\mathrm{GHz})$	$f_{max}\;(\mathrm{GHz})$	Δf(GHz)	Minimum Return Loss (dB)
1	3.38	6.47	3.09	−13.81
	8.55	10.07	1.52	−18.41
	11.02	11.66	0.64	−10.58
	12.28	12.9	0.62	−10.67
	14.11	15.38	1.27	−13.03
	19.76	21.22	1.46	−13.45
	23.7	25	1.3	−16.56
2	8.57	10.04	1.47	−18.96
	10.76	11.42	0.66	−11.97
	12.24	12.94	0.7	−11.16
	14.32	15.38	1.06	−11.91
	19.62	25	5.38	−25.91
3	3.58	6.12	2.54	−12.82
	7.46	7.75	0.29	−12.77
	9.7	10.43	0.73	−16.8
	12.75	16.69	3.94	−17.13
	21.12	23.3	2.18	−15.43
	24.6	25	0.4	−16.04
4	3.75	6.21	2.46	−13.8
	8.56	10.13	1.57	−20.38
	10.65	11.16	0.51	−13.76
	12.26	12.94	0.68	−11.36
	14.06	15.79	1.73	−12.53
	19.82	25	5.18	−26.29
5	3.25	6.67	3.42	−15.97
	8.45	10.11	1.66	−29.33
	10.51	17.06	6.55	−14.29
	19.72	25	5.28	−20.84

**Table 4:** The comparative analysis of the calculated half-power beamwidth (HPBW) for the antennas at both port excitation conditions P_1_ = 1 and P_2_ = 1.

Design	Polarization	HPBW Range (Degree)	HPBW (Degree)
1	Co	−180 to −173	7
		−154 to −110	44
		−45 to −26	19
		−6 to 7	13
		25 to 46	21
		110 to 153	43
		170 to 180	10
	Cross	−63 to −27	36
		129 to 146	17
2	Co	−148 to −106	42
		105 to 149	42
	Cross	−63 to −26	37
3	Co	−134 to −59	75
		62 to 134	72
	Cross	−67 to −30	37
4	Co	−180 to −175	5
		−164 to −104	60
		−50 to −10	40
		16 t0 50	24
		103 to 165	62
		175 to 180	5
	Cross	−126 to −124	2
		−65 to −29	36
		121 to 158	37
5	Co	−180 to −177	3
		−152 to −106	46
		32 to 43	9
		110 to 152	42
		177 to 180	3
	Cross	−64 to −26	38
		133 to 146	13

**Table 5:** Comparative analysis of the proposed MIMO antenna with previously published results in terms of different antenna parameters.

No of Elements	Isolation (in dB)	Design Complexity	Antenna Area (mm^2^)	ECC	Value of Peak Gain (dBi)	Reference
2	25	No	1950	<0.1	>10	This structure
4	21	No	2460	<0.25	6.5	[29]
2	20	No	4371	-	3.5	[35]
4	10	No	2025	<0.25	2.75	[19]
4	17	Yes	1600	0.06	2.9	[36]
4	15	No	1600	0.4	3.5	[37]
2	22	No	640	-	1	[21]
4	10	Yes	5184	<0.014	3.1	[38]
2	21	No	680	-	4	[22]
4	13	No	5624	<0.04	6.2	[39]
2	-	Yes	2880	<0.02	3	[40]
4	13	Yes	5625	<0.04	6.2	[41]
4	15	Yes	16,800	<0.10	5.5	[42]
2	15	Yes	13,125	<0.15	5.1	[43]
4	11	No	1600	<0.1	4	[18]
4	15.4	Yes	676	<0.01	1.41	[20]
4	22	Yes	1750	0.003	-	[44]

## Data Availability

Data are available based upon reasonable request from corresponding author.
